# Anemia in prospective blood donors deferred by the copper sulphate technique of hemoglobin estimation

**DOI:** 10.1186/s12878-015-0035-3

**Published:** 2015-10-22

**Authors:** Samuel Antwi-Baffour, David Kwasie Annor, Jonathan Kofi Adjei, Ransford Kyeremeh, George Kpentey, Foster Kyei

**Affiliations:** Department of Medical Laboratory Sciences, School of Biomedical and Allied Health Sciences, College of Health Sciences, University of Ghana, P. O. Box KB 143, Korle-Bu Accra, Ghana; The Central Medical Laboratories, Korle-bu Teaching Hospital, Accra, Ghana; College of Agriculture and Natural Sciences, School of Biological Sciences, Department of Molecular Biology and Biotechnology, University of Cape Coast, Cape Coast, Ghana

**Keywords:** Anemia, Blood donors, Blood transfusion, Donor deferrals, Hemoglobin

## Abstract

**Background:**

Patients who require transfusion as part of their clinical management have the right to expect sufficient blood to be available to meet their needs and to receive the safest blood possible. Donor deferrals (disqualification) lead to loss of precious blood donors and blood units available for transfusion purposes. It is believed that a large majority of donor deferrals are due to temporal and correctable causes such as anemia in developing countries. It is therefore important to determine anemia among donor population to inform decision-making on the type of measures to be taken to reduce deferrals due to anemia. The aim of the study was to determine anemia in prospective blood donors deferred by the copper sulphate technique of hemoglobin estimation. This, to provide information that would help plan a future strategy for donor recruitment and management.

**Methods:**

Three (3) ml of venous blood samples were collected from the study subjects into EDTA anticoagulant tubes. The hemoglobin levels and red cell indices were measured using Sysmex hematology analyser. A thin blood film was prepared and stained using Leishman stain and then observed under the light microscope.

**Results:**

The prevalence of anemia among the total deferred patients (538) was 17.1 %. Four different types of anemia were found among the subjects. These were normocytic normochromic (46.74 %), microcytic hypochromic (42.39 %) normocytic hypochromic (8.70 %), and microcytic normochromic anemia (2.17 %).

**Conclusion:**

The study showed that a significant number of the prospective blood donors deferred for having low hemoglobin by the copper sulphate method turned out to have anemia by the standard method of diagnosis. Prevalence of anemia among apparently healthy blood donors was therefore higher than expected. Measures must therefore be taken to address this in order not to lose potential blood donors due to a correctable and preventable cause such as anemia.

## Background

Blood transfusion is an essential part of health care. It contributes to saving millions of lives each year in both routine and emergency situations [1]. It permits increasingly complex medical and surgical interventions and dramatically increases the life expectancy and quality of life of patients with a variety of acute and chronic conditions [1, 2]. To make blood transfusion safe for patients, many safety measures are undertaken by the blood transfusion community and the most important is the selection of blood donors [2]. Blood donor suitability standards are based on science, informed medical opinion, and regulatory rules. These are criteria designed to protect both the donor and the recipient [3]. Individuals disqualified from donating blood are known as “deferred” donors and they are deferred for various reasons [3].

Deferrals can be categorized as temporary short term (1–56 days), temporary long term (57–365 days), and multiple years/permanent (more than 365 days) [4]. The key to recruiting and retaining safe blood donors is good epidemiological data on the prevalence and incidence, where possible, of infectious markers in the population which help to identify low-risk donor populations. Also, a satisfying experience during blood donation, good donor care and effective communication between blood donation centre staff and blood donors are all essential factors for the retention of safe blood donors [1]. The deferral of blood donors is a painful and sad experience for the blood donor as well as the blood donation centre screening the donor. These deferrals “bleed” the donor-recruiting efforts of a blood centre, wasting efforts to gain new donors. Moreover, deferring prospective donors often leaves them with negative feelings about themselves as well as the blood donation process [5]. Additionally these donors are less likely to return for blood donation in the future [6].

In developing countries, it is believed that a large majority of donor deferral could be due to a temporal and correctable cause such as anemia which is a condition of less than normal levels of healthy red blood cells circulating in the blood stream [7]. The severity of anemia is measured by a person’s hemoglobin level and the causes could be nutritional (e.g. iron) deficiency, haemorrhage, decreased synthesis of red blood cells or hemoglobin, and so on [8, 9]. Most donor centres use the copper sulphate method to estimate blood levels and defer those who fail the test. However, not all these people may be truly anemic. It is therefore important to determine anemia amongst them using the standard diagnostic method so that those found to be truly anemic could be advised on measures required to treat and prevent the anemia.

The truth is, estimates of anemia prevalence by themselves are only useful if they are associated with a picture of the various causal factors that contribute to the development of the anemia in specific settings [10, 11]. Indeed these factors are multiple and complex and it is critical to collect accurate information about them to provide the basis for developing the best interventions for anemia control [12, 13]. The overall objective of this study was to determine anemia using standard diagnostic methods in prospective blood donors deferred as a result of failing to attain the expected blood levels required using the copper sulphate technique. The outcome is expected to show who among those deferred was truly anaemic and what type of anemia they have. This will help in the planning of future strategies for donor recruitment and management.

## Methods

### Study design

This study was a cross sectional study carried out in June 2012.

### Study population

The subjects were consenting potential blood donors who failed to meet the requirement for donation due to low hemoglobin level using the copper sulphate technique.

### Ethical consideration

Ethical clearance for this research was sought from the Ethics and Protocol Review Committee at the School of Biomedical and Allied Health Sciences, University of Ghana, Legon. All the participants gave their informed consent before their samples were collected*.*

### Materials

Some of the materials used include: Hematology analyser (Sysmex 2000i), Sysmex cell pack and stromatolyser, 5ml K_2_EDTA test tubes and Light Microscope.

### Sample collection and analysis

Three millilitres (3 ml) of blood sample was collected from each of the subject into Ethylene Diamine Tetra-Acetic acid (EDTA) anticoagulant test tube. The samples were then sent to the laboratory within 4 h of collection to be analysed on the hematology analyzer (Sysmex 2000i). Thin blood film was prepared and stained using Leishman stain for morphologic assessment of the red blood cells. The film was then examined under the light microscope using ×40 objective to select a good area for examination and then a drop of oil placed on the film and examined with the ×100 objective.

### Data analysis

The statistical analysis of the data was done using the Statistical Package for Social Sciences (SPSS) version 16.0. Data was represented as frequency Tables and graphs. Pearson’s correlation was used to test the relationship between the various variables that was measured as well as the characteristics of the respondents.

## Results

A total number of 1263 prospective blood donors presented to donate blood during the period of this study. Out of these, 1120 (88.68 %) were males and 143 (11.32 %) females. A total of 538 (42.6 %) involving 444 males and 94 females were deferred due to varied reasons and 114 (21.2 %) of these were due to low hemoglobin (Hb) level. These were assessed for anemia using Hb and red cell indices from a hematology autoanalyser and morphology. Those with Hb < 13.5 for males and Hb < 12.0 for females were termed as anemic. Ninety two (92) forming 80.7 % of the 114 were eventually diagnosed with various forms of anemia using the standard methods (FBC and morphology). This represented 17.1 % of the total number (538) deferred and 7.3 % of the total (1263) prospective donors (Table 1). Twenty two (22) forming 19.3 % of those who failed the copper sulphate test were found not to be anemic.

**Table 1:** A Table of participant’s profiles, mean and standard deviation of the Hb concentrations and the frequencies of the severity of anemias detected

PARAMETERS	MALES	FEMALES	TOTAL
Number of donors	1120 (88.68 %)	143 (11.32 %)	1263
Number of deferrals	444	94	538
Number of deferrals due to low Hb by CuSO_4_	68	46	114
Minimum Hb (g/dl)	7.1	7.7	
Maximum Hb (g/dl)	14.7	12.9	
Mean Hb+/-SD	11.9/1.6	11.0/1.2	
Mild anemia (Hb 10.0–13.5g/dl)	44	33	77
Moderate anemia (Hb 7.0–10.0g/dl)	9	6	15

From the Table, it shows that the number of male donors surpassed that of females by a wide margin, but among those deferred for low Hb, the numbers were not that wide apart. The males also recorded both the lowest and highest Hb levels. It can also be seen that a greater number of participants had mild anemia in comparison to moderate anemia

**Fig. 1:**
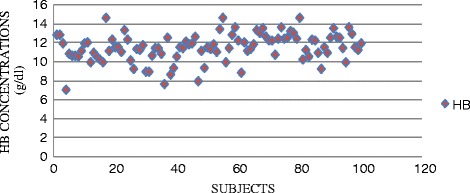
Diagram showing the distribution of hemoglobin concentrations

The mean age of the 114 (68 males and 46 females) deferred donors due to low blood levels was 30 years with majority of them between 21–30 years. The mean Hb concentration and standard deviation of both males and females was 11.5g/dl ±1.5 as well as Frequencies of the severity of anemia in both sexes are all shown in Table 1. The distribution of hemoglobin concentration is shown in Fig. 1.

**Fig. 2:**
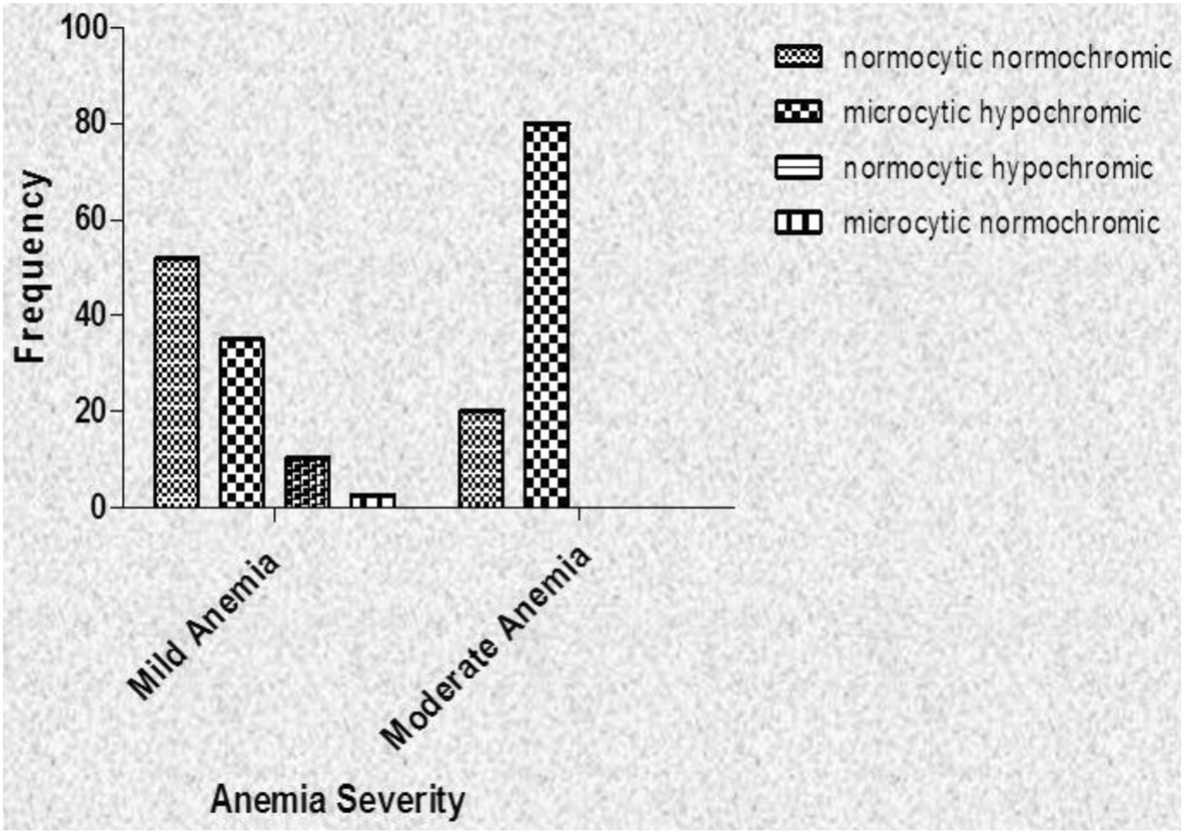
A graph of the distribution of the types of anemia in relation to the severity of the anemia. The participants who were diagnosed with anemia were categorised into mild anemia group with the Hb concentration of 10.0–13.5g/dl and moderate anemia with Hb concentration of 7.0–10.0g/dl. From Fig. 2, it can be seen that majority of those with moderate anemia had microcytic hypochromic anemia with the rest presenting with normocytic normochromic type. On the other hand, those with mild anemia had a fair distribution except for the microcytic normochromic group

Four different types of anemia based on the red cell indices and morphology studies were found in the participants who were diagnosed with anemia. These were normocytic normochromic, normocytic hypochromic, microcytic normochromic and microcytic hypochromic. Comparative analysis of gender and age distribution against the types of anemia was also done and no correlation was established (Table 2). Also analysed was the distribution of the types of anemia in relation to the severity. Majority of the respondents who had mild anemia had normocytic normochromic anemia (51.95 %), while the rest had microcytic hypochromic anemia (35.06 %), normocytic hypochromic anemia (10.39 %) and microcytic normochromic anemia (2.60 %). Majority of those who had moderate anemia had microcytic hypochromic anemia (80.00 %) and the rest had normocytic normochromic anemia (20.00 %) (Fig. 2).

**Table 2:** Distribution of the types of anemia in relation to gender and age of participants

Type of anemia		Normocytic normochromic	Microcytic hypochromic	Microcytic normochromic	Normocytic hypochromic	Total
Sex	Males	27	20	1	5	53
	Females	16	19	1	3	39
Age cate-gory	<20	5	1	1	1	8
	21–25	8	11	1	3	23
	26–30	13	10	0	2	25
	31–35	8	8	0	1	17
	36–40	3	7	0	1	11
	41–45	4	1	0	0	5
	46–50	1	0	0	0	1
	51–55	1	0	0	0	1
	56–60	0	1	0	0	1

It can be seen from the Table that a greater number of the participants presented with Normocytic normochromic, followed by Microcytic hypochromic, Normocytic hypochromic and lastly Microcytic normochromic. Age category of 26–30 had the highest number of anemia cases

## Discussion

In spite of the many programs and interventions undertaken by the various national blood transfusion services, blood transfusion service centres continues to be unsuccessful in their efforts to maintain required stock levels of blood. A lot of potential donors are deferred for reasons which are sometimes temporal (e.g. low Hb level) and majority of these donors do not return to the centre again. This study established that the deferral rate among blood donors was significantly high (42.6 %) of which low hemoglobin levels by CuSO_4_ formed 21.2 %. This rate varies from the findings of a study in India which had total deferral rate of 11.5 % of which 15.5 % was due to anemia which incidentally compares well with the 17.1 % obtained in our study [14, 15].

Our study discovered four types of anemia based on the red cell indices and blood morphology. These were normocytic normochromic, normocytic hypochromic, microcytic normochromic and microcytic hypochromic anemia. The types of anemia with the highest frequency were normocytic normochromic anemia (46.7 %) and microcytic hypochromic anemia (42.4 %). The findings also showed that the prevalence of anemia was significantly higher in female donors than males. Again, 83.7 % out of the 92 diagnosed with anemia presented with mild anemia against 16.3 % with moderate anemia [16]. This means that most of them can have their blood levels improved in a short period of time with proper advice and medication making it possible to return at a later date to donate [17].

Furthermore, statistical analysis (Table 2) showed no relationship between gender and type of anemia (*p* > 0.05), neither was there a relationship between the age and type of anemia (*p* > 0.05). Also, there was no relationship between the ages of the respondents and the severity of anemia (*p* > 0.05) and between the gender of the respondents and the severity of anemia (*p* > 0.05). The lack of relationship between these parameters could be as a result of the fact that the respondents were apparently healthy individuals who had passed the same criteria for selection for donation as healthy individuals prior to blood level estimation. The results of the study however, showed significant relationship between the severity of anemia and the type of anemia (*p* < 0.05).

The findings from our study would help blood collection centres to plan a future strategy for donor recruitment and management by using the standard method to test potential donors who fail the copper sulphate procedure of Hb estimation. This way, people who would be found truly anemic can be assisted to improve their condition with informed advice and medication so they can return to donate. Again, those found to be falsely low can then go ahead and donate. In general, it will help blood collection centres to develop and implement strategies and tactics to manage blood donors well and to evaluate the effectiveness of programs and services as far as donor safety is concern. Subsequently they will be able to support the delivery, sustainment and growth of donor recruitment and retention [18, 19].

Furthermore, the outcome will also assist in formulating a policy guidance on providing blood donor counselling as an essential component of quality donor service and care and as a requirement for a safe blood supply. The counselling of blood donors is an important means of promoting healthy lifestyles and makes an important contribution to individual and community health [20]. In addition, counselling contributes to the early diagnosis and treatment of conditions such as anemia, blood disorders and infections which will offer a crucial early entry point for the treatment and care of donors found to be unfit [21, 22]. Counselling may also reduce adverse donor reactions, improve donor perceptions of the blood transfusion services and, most importantly, increase the likelihood of them returning for future donation [23]. This way, donor deferrals or lost particularly due to anemia may be reduced.

## Conclusion

There was a high prevalence of deferral of prospective blood donors and a significant proportion of these deferred donors were as a result of low Hb levels and subsequently anemia. Normocytic normochromic anemia and microcytic hypochromic anemia were the most common type of anemia detected. Most of them had mild anemia which can be reversed in a short period of time if the necessary advice and treatment is given. It can therefore be concluded that people who fail the copper sulphate procedure of Hb testing should not be just sent way as deferred donors but rather be assessed for anemia and be offered advice and treatment so that if their anemia can be corrected, they are encouraged to come back to donate.

## Abbreviations

CuSO_4_: Copper sulphate
EDTA: Ethylene Diamine Tetra-Acetic acid
FBC: Full Blood Count
Hb: Hemoglobin
RBC: Red Blood Cell
RDW: Red Cell Distribution Width
WBC: White Blood Cell
WHO: World Health Organization
